# Density-Dependent Natal Dispersal Patterns in a Leopard Population Recovering from Over-Harvest

**DOI:** 10.1371/journal.pone.0122355

**Published:** 2015-04-13

**Authors:** Julien Fattebert, Guy Balme, Tristan Dickerson, Rob Slotow, Luke Hunter

**Affiliations:** 1 Panthera, New York, New York, United States of America; 2 School of Life Sciences, University of KwaZulu-Natal, Durban, South Africa; 3 Department of Biological Sciences, University of Cape Town, Cape Town, South Africa; 4 Department of Genetics, Evolution and Environment, University College, London, United Kingdom; University of Tasmania, AUSTRALIA

## Abstract

Natal dispersal enables population connectivity, gene flow and metapopulation dynamics. In polygynous mammals, dispersal is typically male-biased. Classically, the ‘mate competition’, ‘resource competition’ and ‘resident fitness’ hypotheses predict density-dependent dispersal patterns, while the ‘inbreeding avoidance’ hypothesis posits density-independent dispersal. In a leopard (*Panthera pardus*) population recovering from over-harvest, we investigated the effect of sex, population density and prey biomass, on age of natal dispersal, distance dispersed, probability of emigration and dispersal success. Over an 11-year period, we tracked 35 subadult leopards using VHF and GPS telemetry. Subadult leopards initiated dispersal at 13.6 ± 0.4 months. Age at commencement of dispersal was positively density-dependent. Although males (11.0 ± 2.5 km) generally dispersed further than females (2.7 ± 0.4 km), some males exhibited opportunistic philopatry when the population was below capacity. All 13 females were philopatric, while 12 of 22 males emigrated. Male dispersal distance and emigration probability followed a quadratic relationship with population density, whereas female dispersal distance was inversely density-dependent. Eight of 12 known-fate females and 5 of 12 known-fate male leopards were successful in settling. Dispersal success did not vary with population density, prey biomass, and for males, neither between dispersal strategies (philopatry vs. emigration). Females formed matrilineal kin clusters, supporting the resident fitness hypothesis. Conversely, mate competition appeared the main driver for male leopard dispersal. We demonstrate that dispersal patterns changed over time, i.e. as the leopard population density increased. We conclude that conservation interventions that facilitated local demographic recovery in the study area also restored dispersal patterns disrupted by unsustainable harvesting, and that this indirectly improved connectivity among leopard populations over a larger landscape.

## Introduction

In fragmented, human-dominated landscapes, most animal populations persist as sets of geographically discrete populations isolated in a highly altered matrix [1]. In this context, natal dispersal, the permanent emigration from the natal range to an area where an individual settles and breeds [2], is essential to maintain demographic and genetic flow among population patches [3]. Natal dispersal improves the long-term persistence of spatially-structured populations in a metapopulation fashion [4–6]. Human-mediated harvest can elevate the rate of territorial turn-over and provide opportunities for subadults to settle locally, therefore disrupting natal dispersal patterns [7]. Such decreased rate of emigration can turn source populations into sinks [7], or lead to increased inbreeding within the local population [8], consequently affecting the dynamics and persistence of the larger population. Understanding the impacts of anthropogenic disturbance on dispersal patterns is therefore critical to effectively managing harvested populations.

In polygynous mammals, females tend to remain philopatric and natal dispersal is often male-biased [9]. In the absence of male parental care, females invest much more than males in individual offspring, and benefit more from a local knowledge of resources (the 'resident fitness' hypothesis) [10]. Females should therefore compete for philopatry, and breed within or next to their natal range, forming kin clusters and gaining from inclusive fitness [11]. Male-biased dispersal, in contrast, is classically explained by the ‘inbreeding avoidance’, ‘mate competition’ or ‘resource competition’ hypotheses [9, 12, 13]. The ‘inbreeding avoidance’ hypothesis posits that males disperse to avoid inbreeding with related females, resulting in density-independent sex-biased dispersal [14]. The ‘mate competition’ hypothesis, in contrast, suggests that subadult males disperse to avoid competition for mates with conspecific males; hence, dispersal rates and distances should increase at higher male densities [12]. The ‘resource competition’ hypothesis similarly postulates that dispersal is density-dependent but among both sexes, as individuals disperse to avoid competition for limiting resources, particularly food and space (density-dependent dispersal) [15]. Probability of emigration and dispersal distance, however, might also decrease after population density saturates and mate or resource competition with unrelated conspecifics in distant areas becomes too costly (pre-saturation dispersal) [11, 16, 17]. These hypotheses are not mutually exclusive, and the causes for dispersal differ across species and populations [14].

Natal dispersal patterns and mechanisms are poorly studied and understood in large wide-ranging and nocturnal carnivores, particularly in cryptic solitary species such as leopards (*Panthera pardus*) [18]. We used a quasi-experimental design to test density-dependence effects on natal dispersal patterns in a leopard population released from anthropogenically driven demographic decline [19]. Specifically, we assessed the effect of sex, population density and prey biomass on (1) age at commencement of dispersal, (2) distance dispersed, (3) probability of emigration, and (4) success of dispersers to reach breeding age and settle in an independent home-range, in subadult male and female leopards. We discuss these patterns in relation to the ‘inbreeding avoidance’, ‘mate competition’, ‘resource competition’ and ‘resident fitness’ hypotheses, and the conservation implications of the observed dispersal patterns for functional metapopulations over a larger landscape.

## Material and Methods

### Study Area and Study Population

**Fig 1:**
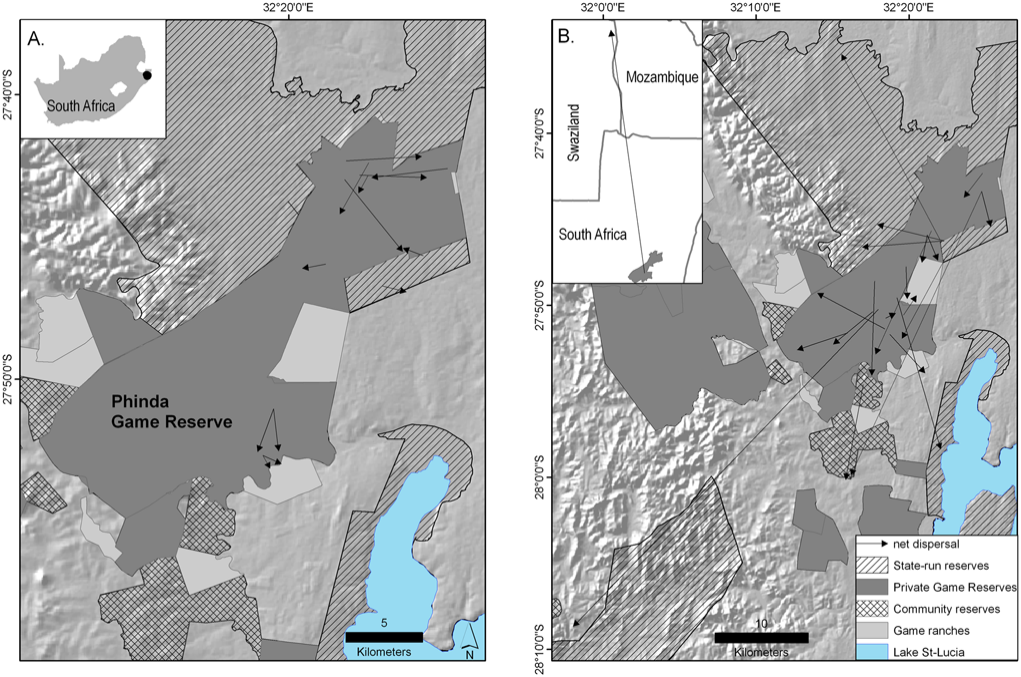
Net dispersal distance of subadult leopards, Phinda Private Game Reserve, South Africa, 2002–2012. (a) Subadult females (n = 13), with inset showing the location of Phinda in South Africa, (b) subadult male leopards (n = 22), with inset showing net long-distance dispersal of male M67 to north-eastern Swaziland.

We studied leopard dispersal ecology in Phinda Private Game Reserve (hereafter Phinda; 234 km^2^; Fig 1) in northern KwaZulu-Natal, South Africa (27° 33–27° 55’ S, 32°06’– 32° 26’ E). Phinda is surrounded by a mosaic of protected and non-protected areas, consisting of private and state-run game reserves, local pastoral communities, livestock ranches, game ranches, and various crop and timber plantations. Leopards are not constrained by boundary fences and move freely across the region [20]. Individuals from Phinda are exposed to greater mortality risk in surrounding non-protected areas, from a combination of legal trophy hunting, legal and illegal problem animal control, and illegal off-take for skins [19, 21]. Although formally protected, Phinda’s leopard population was in decline prior to 2005 (annual population growth rate λ = 0.978) due to high levels of anthropogenic mortality in surrounding non-protected areas (annual mortality rate AMR = 0.401) [19]. Following the implementation of sustainable harvest protocols along with other conservation interventions in 2005, population annual mortality rate declined (AMR = 0.134) and annual growth rate increased (λ = 0.136). By 2009 the leopard population in Phinda reached and stabilized at putative carrying capacity (from 7.2 ± 1.1 to 11.2 ± 2.1 leopards/100 km^2^) [19, 21]. Over the same period, adult leopards in Phinda adjusted their space-use following a dual reproductive strategy; males maintained large home-ranges while female home-range size decreased [22].

### Capture, Handling, and Radio-Tracking

We captured leopards in Phinda between 2002 and 2012, with a combination of free-darting, cage-trapping and soft-hold foot-snaring following Balme et al. [23]. We aged leopards using tooth wear [24] and a combination of morphological cues [25]. We classified leopards into three age classes: cubs <1 year old; subadults 1–3 years old; and adults >3 years old. Depending on their age and the accessibility for radio-tracking (within vs. outside the protected area), leopards >10 months were fitted with a VHF (250 g, Sirtrack Ltd., New Havelock North, Zealand; 0.5% of adult female body mass) or GPS collar (420 g, Vectronic-Aerospace, Berlin, Germany; 1.2% of adult female body mass). We typically fitted subadult males <18 months with a VHF collar and males >18 months with a GPS collar. We equipped subadult male collars with a drop-off mechanism (c. 50 g Sirtrack Ltd., Havelock North, New Zealand) set to release 6–12 months after deployment [18]. We located VHF-collared individuals on average every three days from the ground by homing-in or radio-triangulation. GPS collars were programmed to acquire 2–6 fixes daily (mean fix success rate: 0.795 ± 0.035 (SE), n = 28 collars). GPS data were screened for potentially large locational errors by removing 3D fixes with PDOP >15 and 2D fixes with PDOP >5 [26].

### Natal and Settling Home-Ranges

We defined the natal range of subadult leopards of known origin (i.e. born in the study population to telemetered females) as the annual home-range used by their mother during their first year. Successful dispersal typically entails establishing an independent home-range and breeding [2]. Without proof of breeding for most males, we assumed subadult leopards to be successful dispersers when they used the same home-range for a minimum of six months, or when they reached 3 years old [7]. We used Geospatial Modelling Environment (GME) [27] to compute the 95% isopleths fixed-kernel home-ranges [28]. We calculated the bandwidth of the smoothing factor *h* using the ‘solve the equation plug-in’ method [29].

### Dispersal Parameters

#### Dispersal age

We defined age at commencement of dispersal for known-origin individuals as the age (in months) that they took their first exploratory foray out of their natal range as nutritionally independent subadults.

#### Dispersal distance

We measured net dispersal distance as the Euclidean distance between the origin and the final location of subadult individuals [7]. We defined origin as the centroid of the natal range for individuals of known origin, or the capture site for subadult individuals of unknown origin. We defined final location as the centroid of their permanent home-range for individuals that settled. For individuals that died, or for which contact was lost before they reached 3 years old, we defined final location as the location of death, or the last recorded location respectively [7]. As these individuals might have dispersed further than recorded, these distances should be considered as a minimum [7, 30].

#### Emigration

A large proportion of the individuals was of unknown origin (43%), or disappeared before they reached adulthood and settled (31%). Therefore, we did not use the classical definition of dispersal and philopatry using a threshold 5% overlap between independent and natal home-ranges [31]. Instead, we classified subadult leopards as ‘emigrant’ or ‘philopatric’ according to their net dispersal distance scaled to the diameter of a circular home-range equivalent in size to the mean annual adult home-range [7]:

$$scaled\;dispersal\;distance\;=\;\frac{net\;dispersal\;distance}{2\sqrt{home-range\;area/\pi }}$$ (1)

We classified individuals that dispersed further than one average adult female home-range diameter as ‘emigrants’ (scaled distance >1), and others as ‘philopatric’ (scaled distance <1). We also scaled net dispersal distance to adult male home-range diameter, to assess dispersal out of the putative paternal range and mate competition avoidance in subadult males. Home-range sizes were taken from Fattebert [22].

### Leopard and Ungulate Biomass Density Estimates

**Fig 2:**
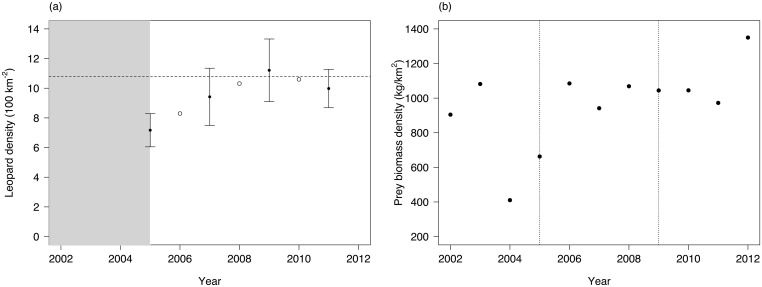
Leopard density and prey biomass estimates, Phinda Private Game Reserve, South Africa, 2002–2012. (a) Photographic capture-recapture leopard density estimates (solid black dots) with standard error and interpolated values (open circles). Grey zone indicates disturbance period when leopards in Phinda GR underwent negative population growth, before the implementation of conservation protocols in 2005. Horizontal dashed line indicates putative carrying capacity at mean leopard density at the core of the population [21]; (b) combined yearly estimates of nyala, impala and warthog biomass derived from aerial count data in Phinda GR, 2002–2012. Fine-dotted lines show in 2005 the implementation of conservation protocol following leopard population decline, and in 2009 the time when the leopard population reached putative carrying capacity [21].

We estimated leopard population density in Phinda in 2005, 2007, 2009, and 2011 using data from camera-trap surveys in closed-population capture-recapture models (Fig 2a; see [32] for details on survey design and analysis). We calculated yearly biomass density estimates for prey using aerial count data collected in Phinda from 2002–2012 (mean species weights from [33]), assuming a monotonic relationship between detectability and density [34]. We combined biomass density estimates of the three main prey species in leopard diet in Phinda [23], namely nyala (*Tragelaphus angasii*), impala (*Aepyceros melampus*) and warthog (*Phacochoerus africanus*) (Fig 2b).

### Statistical Analyses

We tested effects of covariates on dispersal parameters using a generalized linear model (GLM) framework. We screened variables for collinearity using a cut-off of |r| = 0.75. For each dispersal parameter, we built a set of candidate models to explore the effect of the independent variables and their combinations without interaction. We used AIC model selection criteria corrected for sample sizes (AICc) to select the most parsimonious models [35]. When candidate models were within ΔAICc <2, we performed model averaging to estimate unbiased parameter coefficients. Parameter coefficients were deemed significant when the corresponding 90% confidence interval (CI) did not include zero [36].

We tested for differences in dispersal age between sexes. As population density was not assessed every year but was closely related to time (Spearman rank correlation, r_s_ = 0.750; p = 0.033), we used time as a proxy for population trend to test for a density-dependent response. To disentangle the effects of population density from resource availability, we included prey biomass density as a factor in the model to account for annual variability in prey estimates (Fig 2b).

We tested for differences in net dispersal distance between sexes. To meet statistical assumptions we removed one extreme outlier male long-distance dispersal (194.5 km) presented elsewhere [18], and we used log-transformed net dispersal distance data. We tested for changes in net dispersal distance over time separately for each sex. We explored a quadratic response to time on dispersal distance to test for pre-saturation dispersal effect, and we included prey biomass as a factor in the model.

As all females were philopatric (see Results), we only tested for changes in the probability of emigration in subadult males over time, using a GLM with a binomial error structure (logistic regression). We included prey biomass as a factor in the model, and again we explored a quadratic response to time on probability of emigration to test for pre-saturation dispersal effect.

We used a logistic regression to test the effect of time and prey biomass on the likelihood of known-fate subadult leopards to settle successfully, assessing males and females separately. For males, we additionally tested the effect of dispersal strategy (i.e. philopatry *vs*. emigration) on dispersal success. We censored individuals with which contact was lost (i.e., unknown fate).

We ran all statistical tests in R version 3.0.0 [37]. Model averaging was performed using package *MuMIn* [38]. When both linear and quadratic responses to time were within ΔAICc <2, we only used the response with the lowest AICc in the model-averaging process. We report mean *±* standard error (SE) unless otherwise stated.

### Ethic statement

The animal handling procedures for this study were approved by the Animal Ethics Subcommittee of the University of KwaZulu-Natal Ethics Committee (approval 051/12/Animal). Leopards are protected in South Africa and research permission to conduct research on state-protected and private lands was provided by Ezemvelo KwaZulu-Natal Wildlife (permit number 104HO/4004/07).

## Results

Fifty-four leopards (35 males, 19 females) were captured in Phinda between 2002 and 2012. Thirty-five individuals (22 males, 13 females) were tracked during dispersal age, of which 20 were of known origin (11 males, 9 females; Table 1). Twenty-six subadults were fitted with a VHF collar only (13 males, 13 females), and 9 subadult males where fitted with a GPS collar. Eleven subadults died (7 males, 4 females), and contact was lost with a further 11 (10 males, 1 female) before they reached 3 years old. Thirteen subadult leopards settled or survived to 3 years old and were considered successful dispersers (5 males, 8 females).

**Table 1:** Summary statistics of the dispersal parameters for 35 subadult leopards in Phinda Private Game Reserve, South Africa, 2002–2012.

Dispersal parameter	Males	Females
**Origin (n individuals)**		
Known	11	9
Unknown	11	4
**Mean dispersal age** ^**a**^ **(months)**	13.6 ± 0.3 (SE)	13.7 ± 1.0 (SE)
**Mean net dispersal distance** ^**b**^ **(km)**	11.0 ± 2.5 (SE)	2.7 ± 0.4 (SE)
**Dispersal strategy** ^**c**^ **(n individuals)**		
Philopatry	10	13
Emigration	12	-
**Fate (n individuals)**		
Settled	5	8
Dead	7	4
Contact lost	10	1

^a^ Individual of known origin

^b^ One extreme male outlier (M67, 194.5 km) censored from the distance analyses

^c^ Emigrants are individuals that disperse further than one average female home-range diameter (6.1 km)

### Dispersal age

**Fig 3:**
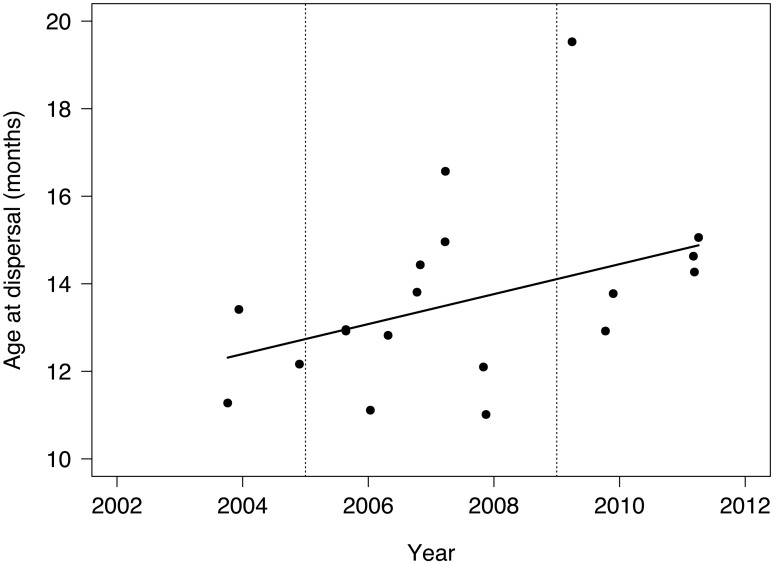
Age at commencement of dispersal in subadult leopards in Phinda Private Game Reserve, South Africa, 2002–2012. There was no significant difference in age at dispersal between sexes, and solid line indicates linear regression fitting all the data (R^2^ = 0.118, F_1,18_ = 3.534, p = 0.076). Fine-dotted lines show in 2005 the implementation of conservation protocol following leopard population decline, and in 2009 the time when the leopard population reached putative carrying capacity [21].

Male (13.6 ± 0.3, range 12.2–15.1 months; n = 11) and female (13.7 ± 1.0, range 11.0–19.5 months; n = 9) leopards generally commenced dispersal at similar ages (Table 1). Subadults typically dispersed at older ages as time progressed (β = 0.3; 90% CI 0.003, 0.7); i.e. as the leopard population density increased (Table 2; Fig 3).

**Table 2:** *A priori* linear models exploring the effect of sex, time as a proxy for population density and prey biomass density on age at commencement of dispersal in subadult leopards in Phinda Game Reserve, South Africa, 2002–2012.

Model	Model parameters	AICc	ΔAICc	w
Dispersal age	**Density**	**87.17**	**0**	**0.37**
	**null**	**87.96**	**0.79**	**0.25**
	Sex + Density	89.29	2.12	0.13
	Prey biomass^a^	90.22	3.06	0.08
	Density + Prey biomass	90.27	3.10	0.08
	Sex	90.74	3.57	0.06
	Sex + Density + Prey biomass	92.76	5.59	0.02
	Sex + Prey biomass	93.36	6.24	0.02

AICc = Akaike Information Criteria adjusted for small sample sizes; ΔAICc = (AICc)–(AICc)_min_; w = Akaike weight. Candidate models with ΔAICc < 2 (bold face) were used for model-coefficient averaging.

^a^ Combined nyala, impala and warthog relative biomass density estimates derived from aerial count data in Phinda GR, 2002–2012.

### Dispersal Distance

**Fig 4:**
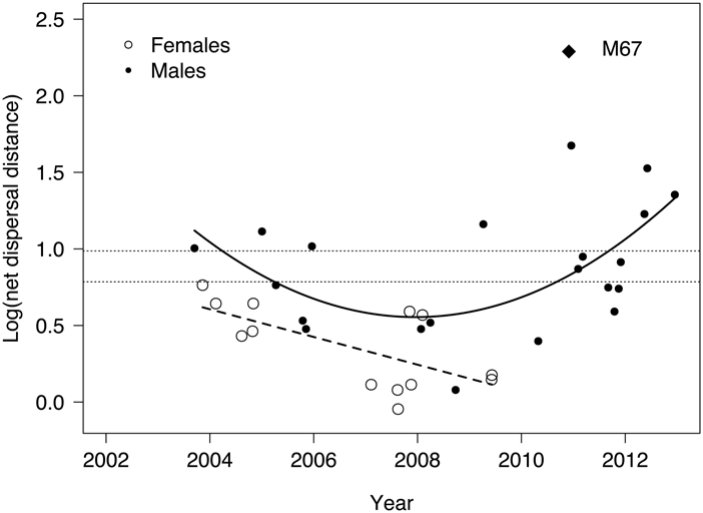
Log-transformed net dispersal distance in subadult female and male leopards in Phinda Private Game Reserve, South Africa, 2002–2012. Dashed line shows a significant linear regression fitting the female data (R^2^ = 0.392, F_1,11_ = 8.728, p = 0.013), solid black curve shows a significant quadratic relationship in the male data (R^2^ = 0.250, F_1,18_ = 3.247, p = 0.029) after removal of one extreme outlier (M67, diamond). Horizontal fine-dotted lines demark the diameter of one average adult female (6.1 km) and male (9.7 km) home-range. Dispersal beyond one adult female home-range diameter defines emigration. Subadult males dispersing further than one adult male home-range diameter escaped mate competition with their putative father.

Males (11.0 ± 2.5, range 1.2–47.3 km; n = 21) generally dispersed further than females (2.7 ± 0.4, range 0.9–5.8 km; n = 13; Table 1; Fig 1). Female dispersal distance decreased linearly with density (β = -0.09; 90% CI -0.1, -0.04; Table 3; Fig 4). Male dispersal distance, in contrast, followed a quadratic relationship with density (linear term: β = -125.1; 90% CI -206.1, -4.4; quadratic term: β = 0.03, 90% CI 0.01, 0.05). The point of inflection in 2008 suggested that male dispersal distance increased three years after the implementation of conservation interventions, once leopard population density had increased to near capacity.

**Table 3:** *A priori* linear models exploring the effect of sex, time as a proxy for population density and prey biomass density on dispersal distance in subadult leopards in Phinda Game Reserve, South Africa, 2002–2012.

Model	Model parameters	AICc	ΔAICc	W
Dispersal distance	**Sex**	**30.76**	**0.00**	**1.00**
	null	42.24	11.48	0.00
Female dispersal distance	**Density (linear)**	**2.96**	**0**	**0.63**
	Density (quadratic)	5.84	2.88	0.15
	Density (linear) + Prey biomass	6.87	3.91	0.09
	null	7.08	4.13	0.08
	Prey biomass	8.54	5.59	0.04
	Density (quadratic) + Prey biomass	11.13	8.17	0.01
Male dispersal distance	**Density (quadratic)**	**22.00**	**0**	**0.48**
	Prey biomass^a^	24.33	2.32	0.15
	null	24.41	2.41	0.15
	Density (quadratic) + Prey biomass	25.28	3.28	0.09
	Density (linear)	25.35	3.35	0.09
	Density (linear) + Prey biomass	27.33	5.32	0.03

AICc = Akaike Information Criteria adjusted for small sample sizes; ΔAICc = (AICc)–(AICc)_min_; w = Akaike weight. Candidate models with ΔAICc < 2 (bold face) were selected as final models.

^a^ Combined nyala, impala and warthog biomass density estimates derived from aerial count data in Phinda GR, 2002–2012.

### Probability of Emigration

All subadult females dispersed less than one average adult female home-range diameter (6.1 km), and were thus considered philopatric (Fig 4). The only female that nearly emigrated (F10) was killed before reaching 3 years old. Twelve of 22 males emigrated further than one average adult female home-range diameter. Consistent with net dispersal distance, the probability of male emigration followed a quadratic relationship with density (linear term: β = -704.0; 90% CI -1404.4, -3.6; quadratic term: β = 0.2; 90% CI 0.001, 0.3; Table 4). In the final averaged model, prey biomass was not a strong predictor of emigration probability (β = 0.003; 90% CI -8.6, 7.1). Of the 12 males that emigrated, nine dispersed further than one adult male home-range diameter (9.7 km; Fig 4).

**Table 4:** *A priori* binomial regression models exploring the effect of time as a proxy for population density and prey biomass density on the probability of dispersal in subadult male leopards in Phinda Private Game Reserve, South Africa, 2002–2012.

Model	Model parameters	AICc	ΔAICc	w
Probability of male emigration	**null**	**32.52**	**0**	**0.27**
	**Density (quadratic)**	**32.64**	**0.12**	**0.25**
	**Prey biomass** ^**a**^	**32.9**	**0.38**	**0.22**
	Density (linear)	34.17	1.66	0.12
	Density (linear) + Prey biomass	34.92	2.41	0.08
	Density (quadratic) + Prey biomass	35.57	3.05	0.06

AICc = Akaike Information Criteria adjusted for small sample sizes; ΔAICc = (AICc)–(AICc)_min_; w = Akaike weight. Candidate models with ΔAICc < 2 (bold face) were used for model-coefficient averaging.

^a^ Combined nyala, impala and warthog biomass density estimates derived from aerial count data in Phinda GR, 2002–2012.

### Success of Dispersal

Of 12 known-fate females, eight successfully established a permanent philopatric home-range, leading to the formation of matrilineal kin clusters (S1 Fig). Female dispersal success did not vary with density or with prey biomass (Table 5). Five of 12 known-fate males were successful in establishing independent home-ranges. Male dispersal success also did not vary with density, prey biomass, or between dispersal strategies (i.e. philopatry vs. emigration).

**Table 5:** *A priori* binomial regression models exploring the effect of time as a proxy for population density and prey biomass density on the success of dispersal in known-fate subadult leopards in Phinda Private Game Reserve, South Africa, 2002–2012.

Model	Model parameters	AICc	ΔAICc	w
Female success	**null**	**17.68**	**0**	**0.62**
	Prey biomass	20.13	2.45	0.18
	Density	20.52	2.84	0.15
	Density + Prey biomass	22.78	5.10	0.05
Male success	**null**	**18.70**	**0**	**0.49**
	Prey biomass^a^	20.87	2.17	0.17
	Dispersal strategy^b^	21.29	2.59	0.13
	Density	21.33	2.62	0.13
	Prey biomass + Dispersal strategy	24.50	5.80	0.03
	Density + Prey biomass	24.52	5.82	0.03
	Density + Dispersal strategy	24.75	6.05	0.02
	Density + Prey biomass + Dispersal strategy	29.20	10.50	0.00

For males, the effect of dispersal strategy was also tested. AICc = Akaike Information Criteria adjusted for small sample sizes; ΔAICc = (AICc)–(AICc)_min_; w = Akaike weight. Candidate models with ΔAICc < 2 (bold face) were selected as final models.

^a^ Combined nyala, impala and warthog biomass density estimates derived from aerial count data in Phinda GR, 2002–2012.

^b^ Philopatry or emigration.

## Discussion

Our findings are consistent with the general pattern of sex-biased natal dispersal described for most polygynous mammals [9], and with patterns of male-biased dispersal previously reported in leopards [39]. All females were philopatric, leading to the formation of matrilineal kin clusters. Similar matrilineal assemblages have been observed in pumas (*Puma concolor*) [40], tigers (*Panthera tigris*) [41–43], and brown bears (*Ursus arctos*) [44]. Such behaviour generally supports the resident fitness hypothesis, when related females tolerate the costs of increased resource competition due to the benefits they gain from inclusive fitness [10, 11]. Adult female home-range size in our study area decreased following the population’s release from anthropogenic perturbation after 2005 [22]. This is likely driven by female philopatry, with mothers contracting and shifting their home-ranges to accommodate daughters. However, mothers can only reduce their home-ranges to a point, at which time female offspring will likely be forced to disperse. Despite the increase in leopard population density post intervention, this point has apparently not been reached during our study [19, 22].

In contrast to females, male leopard dispersal followed a quadratic density-dependent relationship with time, with no evidence of pre-saturation dispersal [16]. Rather, opportunistic male philopatry was documented prior to population saturation, with both dispersal distance and probability of emigration inversely density-dependent before 2008. Male dispersal distance and emigration increased once the estimated population density reached capacity and the socio-spatial organization in adult males stabilized, with less turn-over and fewer territorial vacancies [19, 22]. Such population stability could also explain the later age at commencement of dispersal, with fewer unfamiliar males evicting subadults following territorial take-over [45]. These patterns are generally consistent with the mate competition hypothesis, which predicts density-dependent costs of competition, and increased dispersal rate and distance under higher population density [15]. Although it is difficult to disentangle the relative importance of the different causes for dispersal, the patterns we observed suggest that inbreeding avoidance is more likely a consequence of dispersal under mate competition, rather than the ultimate cause for dispersal in male leopards [46]. When the situation allowed, male leopards remained in their natal ranges; even though this increased the likelihood of breeding with a relative (at least one male was observed mating with his mother; T. Dickerson & G. Balme, pers. obs.). There is also little support for the resource competition hypothesis [13, 15], as prey biomass was not a strong predictor for any of the dispersal parameters that we assessed. However, the year-to year variability observed in prey biomass might have little demographic reality and be due to variability in detectability when performing aerial total counts [47]. Ground-based strip count data partly collected during the duration of our study showed no signification variation of the leopard prey biomass between 1992–1995 and 2003–2005 [23]. Most emigrant subadult males dispersed further than one male home-range diameter and thereby escaped competition with their putative father once the population reached putative capacity. As such, mate competition appears the main driver for male leopard dispersal [12].

Opportunistic philopatry we observed in male leopards could increase the risk of inbreeding [48], particularly as females were also philopatric. Hence, daughters may potentially settle and reach sexual maturity in their fathers’ home-ranges [49]. In well-protected, stable leopard populations, females typically only conceive for the first time at roughly four years, once their father has been, or is near to eviction [19, 50]. However, age at first parturition appears younger in lower-density, disturbed leopard populations, averaging closer to three years, potentially increasing the risk of inbreeding (although male tenure is also often shorter in disturbed populations [19]). In a heavily harvested puma population in Montana, USA, a third of pairings were likely between half-siblings or closer [8].

The short dispersal distances and opportunistic male philopatry documented in our study supports the view that felids are conservative dispersers, especially in comparison to canids or ursids which are more effective colonizers of distant, vacant habitats [30]. However, functional metapopulation dynamics require inter-patch dispersal [5, 51], which appears rare among leopards. Although a number of individuals dispersed successfully, only one subadult male (M72) reached adulthood in a disjoint patch. This may have long-term implications for the genetic diversity of leopards, particularly as our study population displayed characteristics of a sink population before the implementation of conservation interventions [19]. Many subadult male leopards did not get the opportunity to disperse, as they died while still in their natal range. Generally, the poor dispersal abilities of solitary felid populations may increase their risk of extinction [52, 53].

Exclusive philopatry among female leopards may also limit the potential rescue effect of dispersal if the nuclear gene flow is limited to males [5, 52]. Further investigations are needed to understand the patterns of female dispersal over a larger landscape. Population genetic studies could help quantify flow that is challenging to detect through movement studies when successful dispersers make up such a small portion of the subadult cohort [51]. Molecular studies may also provide insight on the genetic impacts that over-harvesting has on dispersal behaviour [8]. Finally, landscape structure can affect the likelihood of emigration, dispersal path, and success of dispersal [54]. Therefore, research on habitat use during dispersal is valuable to better understand the ability of dispersing felids to reach distant populations [20, 52, 55].

### Conclusions

We demonstrate that dispersal patterns changed over time, i.e. as the leopard population density increased. Resident fitness likely explained matrilineal cluster formation and continuous female philopatry. While males could display opportunistic philopatry in an unstable population, mate competition was likely the main driver of male dispersal, with both the rate of male emigration and the distance they dispersed increasing as the population saturated. We earlier showed that leopard population demographic recovery was a consequence of the release from anthropogenic harvest pressure [19, 21]. Hence, we conclude that these conservation interventions not only facilitated local demographic recovery in Phinda, but also indirectly contributed to improve connectivity among leopard populations over a larger landscape through re-establishing dispersal patterns disrupted by unsustainable harvesting. While male leopard dispersal behaviours have been restored, no female dispersed and the female socio-spatial structure had not fully stabilized by the end of the study, seven years later [22]. This highlights the durability of local disturbance, even for resilient species such as the leopard. In order to increase tolerance for the presence of large carnivores outside of protected areas, and in particular for transient, dispersing individuals, we suggest more widespread adoption and strict enforcement of science-based protocols for sustainable legal harvesting of leopards and of livestock protection schemes [19]. This will ensure sufficient demographic and socio-spatial stability of source populations for inter-population dispersal and help safe-guard against further leopard population declines.

## Supporting Information

S1 Fig Example of the establishment of a philopatric subadult female (F15), leading to the formation of a matrilineal cluster in Phinda Private Game Reserve, 2002–2006. Grey polygon shows F15’s annual natal range between 1 October 2002–30 September 2003 (a). Black lines delineate 95% isopleths kernel home-range of mother F11 (solid), female F15 (dashed) and female sibling F16 (dotted), born in October 2002. Subsequent panes (b-f) represent consecutive 6-month time windows. Sibling F16 died before 2 years old. As F15 grew older, F11 shifted to the North and relinquished the southern portion of her initial range to F15 (coordinates are in meters, UTM WGS84 36S).
(TIF)
