# Supplementary figures and images for: Density-Dependent Natal Dispersal Patterns in a Leopard Population Recovering from Over-Harvest
Source: PLoS One. 2015 Apr 13;10(4):e0122355. doi: 10.1371/journal.pone.0122355 (PMC4395424; doi:10.1371/journal.pone.0122355)

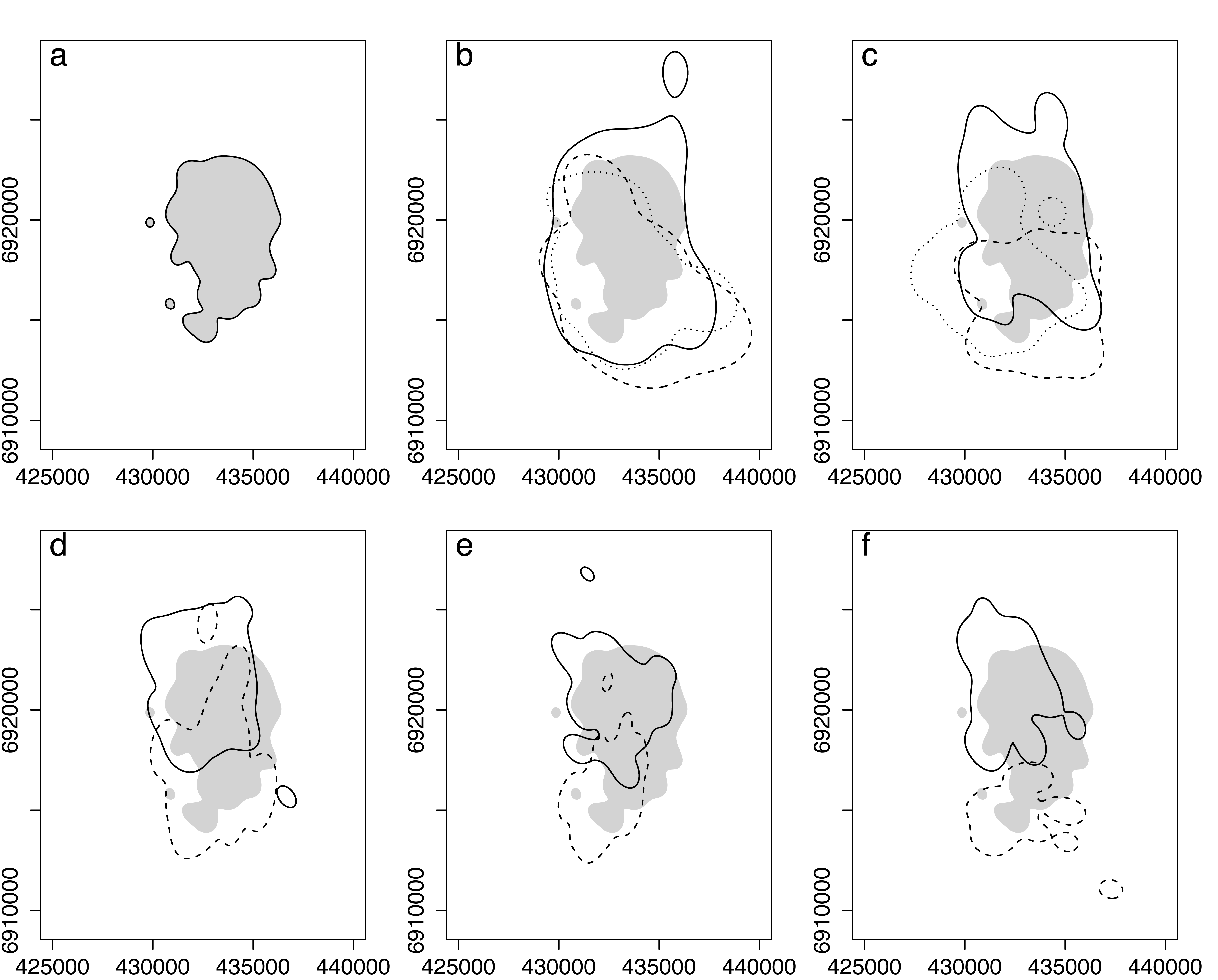

Supplement: S1 Fig — Grey polygon shows F15’s annual natal range between 1 October 2002–30 September 2003 (a). Black lines delineate 95% isopleths kernel home-range of mother F11 (solid), female F15 (dashed) and female sibling F16 (dotted), born in October 2002. Subsequent panes (b-f) represent consecutive 6-month time windows. Sibling F16 died before 2 years old. As F15 grew older, F11 shifted to the North and relinquished the southern portion of her initial range to F15 (coordinates are in meters, UTM WGS84 36S). (TIF) [file pone.0122355.s001.tif]
